# Post-traumatic stress disorder in living donors after pediatric liver transplantation: A cross-sectional investigation study

**DOI:** 10.1097/MD.0000000000015565

**Published:** 2019-05-17

**Authors:** Yimao Zhang, Junxiang Wang, Shuguang Jin, Bo Xiang, Jiaying Yang, Kewei Li, Bing Huang, Wei Lai, Lunan Yan, Jichun Zhao

**Affiliations:** a Department of Pediatric Surgery; b Department of Liver Surgery; c Department of Vascular Surgery; d Department of Intensive Care Unit, West China Hospital of Sichuan University, Chengdu City, Sichuan Province, China.

**Keywords:** living donor, pediatric liver transplant, post-traumatic stress disorder, quality of life, SF-36

## Abstract

Liver transplantation can lead to post-traumatic stress disorder (PTSD) in recipients, but the risk factors associated with PTSD in living donors are unknown. To investigate this progression in pediatric living donors, a cross-sectional investigation was carried out.

All participants completed 2 questionnaires: a PTSD self-rating scale (PTSD-SS) and a validated Chinese version of the Medical Outcomes Study Short Form-36 (SF-36). Clinical and demographic data were collected from medical records and self-report questionnaires. Univariate analysis was conducted to identify statistical differences.

The prevalence of full PTSD (all symptom clusters) and partial PTSD (2 out of 3 symptom clusters) was 12.1% and 31.1%, respectively. Those with an educational status of elementary school (*P* = .001), who were donors to their children (*P* = .008), who were in the first 6 months after transplant (*P* < .001), or were involved in transplants where the recipients had severe complications (*P* = .02) were more likely to have higher PTSD-SS scores than other groups. The non-PTSD group had a higher health-related quality-of-life score compared with the full and partial PTSD groups in the domains of physical function, role-physical, bodily pain, general health, vitality, social functioning, role-emotional, and mental health. In addition, the occurrence of PTSD was related to a poorer quality of life.

The occurrence of PTSD was common in living donors after pediatric liver transplantation. Those with a lower educational status, who were donors to their children, were in the first 6 months after transplant, or were involved in transplants where the recipients had severe complications were most likely to experience PTSD. Post-traumatic stress symptom severity was significantly associated with a poorer quality of life after transplant.

## 1 Introduction

Post-traumatic stress disorder (PTSD) is a debilitating mental disorder related to directly experiencing or witnessing a traumatic, tragic, or terrifying event.^[1,2]^ People with PTSD usually have persistent frightening thoughts and memories of the ordeal and feel emotionally numb.^[3,4]^ While many of those who experience traumatic events can recover well without treatment, if the biological and adaptive coping responses are inadequate or the stress becomes overwhelming, psychological distress can intensify over time and eventually lead to PTSD. Surgical innovations and improvements in immunosuppressants have radically changed the prognosis for solid organ transplant recipients in recent years. This is also true of pediatric liver transplantation, for which outcomes and prognoses have improved.^[5]^ Despite these advances, however, liver transplantation still has a huge physical and psychological impact on recipients and living donors. We have previously reported on PTSD in adult liver transplant recipients.^[6]^ In this study, we aim to report on PTSD, including prevalence, potential risk factors, and self-reported health-related quality of life (HRQoL), in living donors after pediatric liver transplantation.

## 2 Methods

### 2.1 Participants

From January, 2008 to August, 2018, 154 consecutive living donors for pediatric liver transplantation at West China Hospital of Sichuan University were approached for participation. The investigation extended from July, 2017 to August, 2018. Inclusion criteria were: aged over 18 years and with a postsurgery period over 12 months. The exclusion criterion was severe medical complications that prevented the donors from completing the questionnaires or limited their expression. Donors who were lost to follow-up were also excluded from this study. All participants were required to complete a PTSD self-rating scale (PTSD-SS) and a validated Chinese version^[7]^ of the Medical Outcomes Study Short Form-36 (SF-36).^[8,9]^ Questionnaires were completed by interview or mail. Clinical and demographic data were collected from medical records and self-report questionnaires.

### 2.2 Instruments

Post-traumatic stress disorder is an anxiety disorder characterized by 3 general symptom clusters: re-experiencing traumatic events; avoidance of people, places, or things that serve as reminders of the trauma; and chronic hyperarousal symptoms. The PTSD-SS is a 24-item self-reported inventory in which each item is rated on a 5-point scale of distress (1–5) ranging from “not at all” to “extremely” distressing. Moreover, PTSD can be categorized into “full PTSD” or “partial PTSD.” PTSD in participants who scored a minimum of 1 on re-experiencing symptoms, 3 on avoidance symptoms, and 2 on hyperarousal symptoms at a severity level of at least 3 (midrange) per item were defined as having full PTSD. PTSD in participants who experienced 2 of the 3 symptom clusters was defined as partial. Total symptom severity of post-traumatic stress was measured by summing the severity ratings of individual items. The internal consistency (Cronbach α), and split-half and test-retest reliability coefficients were 0.9207, 0.9539, and 0.8677, respectively. The validity of the PTSD-SS was confirmed.^[10]^

Health-related quality of life was assessed by the Chinese version of the SF-36^[7,8,9]^—a valid and self-administered questionnaire used internationally to measure 8 major health concepts: physical function, role-physical, bodily pain, general health, vitality, social functioning, role-emotional, and mental health over 12 months. Role-physical condition included limit in time of tasks, accomplishment with tasks, limit types of tasks, and difficulty with tasks. The raw scores of each subscale were transformed into scores that ranged from 0 to 100. Higher scores indicated higher levels of function or well-being.

### 2.3 Ethical considerations

The study protocol conformed to the ethical guidelines of the 1975 Declaration of Helsinki and was approved by the Ethics Committee of West China Hospital of Sichuan University. All participants provided written informed consent.

### 2.4 Statistical analysis

Statistical analysis was performed by SPSS 25 statistical software. Between-group differences were tested by the independent-samples Student *t* test, analysis of variance, or nonparametric test, as appropriate. Multiple comparisons of observed means were performed by the Student–Newman–Keuls test when equal variances could be assumed and by the Games–Howell procedure when they could not. A probability value of *P* < .05 represented a statistically significant difference.

## 3 Results

### 3.1 Donor characteristics

Among the total pediatric liver transplant donors, 14 were lost to follow-up, and 8 were unable to read Chinese. Finally, 132 donors (85.7%) were included in this study. The results of the PTSD-SS and SF-36 questionnaires completed by interview or mail were not significantly different. The mean age of the donors was 29.4 ± 8.0 years. The demographic and transplant-related characteristics of the study population are shown in Table 1.

**Table 1:**
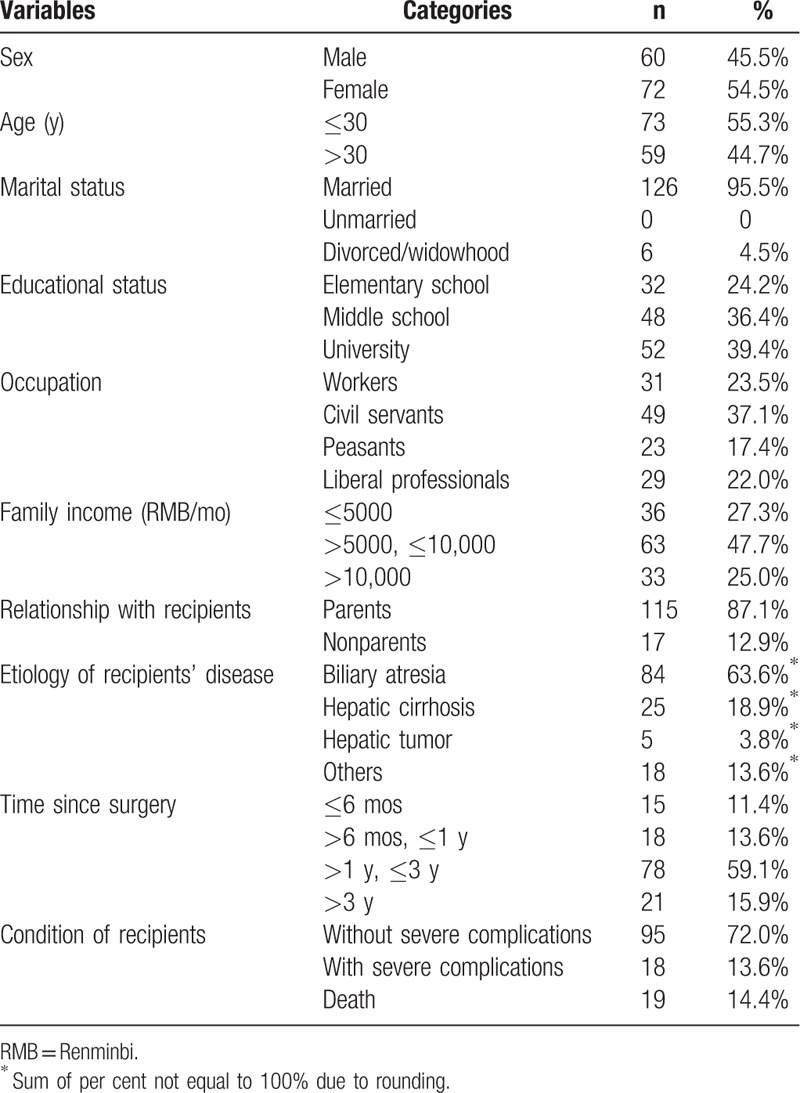
Donors characteristics.

### 3.2 Prevalence of PTSD

The prevalence of PTSD was as follows: 75 cases (56.8%) did not meet the criteria for partial or full PTSD after liver transplant, 41 (31.1%) met the criteria for partial PTSD, and 16 (12.1%) met the criteria for full PTSD.

**Figure 1:**
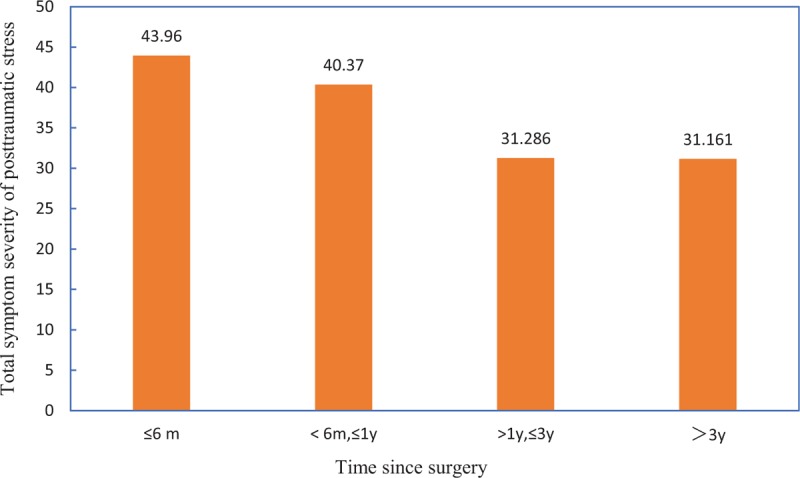
Severity of posttraumatic stress in different stages after surgery.

The results of analysis of posttraumatic stress are shown in Table 2. There were significant differences in donors in terms of educational status (*P* = .001), relationship with recipients (*P* = .008), time since transplant (*P* < .001), and condition of recipients (*P* = .02). Those with an educational level of elementary school, who were donors to their own children, were in the first 6 months after transplant, and were involved in transplants where the recipients suffered severe complications were more likely to have a high PTSD score than other groups. According to Fig. 1, the severity of post-traumatic stress in living donors decreased with time after surgery.

**Table 2:**
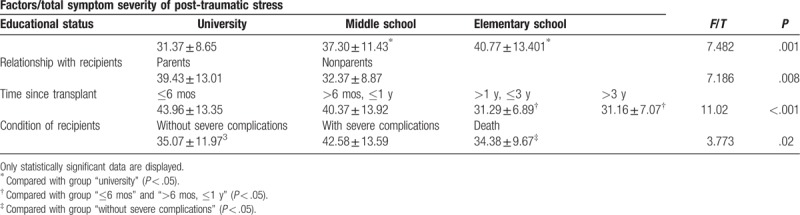
Results of analysis of post-traumatic stress.

The results of the analysis of HRQoL in transplant donors are shown in Table 3. The non-PTSD group had a higher score compared with full and partial PTSD groups in physical function, role-physical, bodily pain, general health, vitality, social functioning, role-emotional, and mental health. There were statistically significant differences between the non-PTSD and full or partial PTSD groups in the role-physical (*P* < .001), general health (*P* = .001), vitality (*P* = .008), social functioning (*P* = .001), role-emotional (*P* < .001), and mental health (*P* = .001) domains. There were also statistically significant differences in the role-emotional domain in the full and partial PTSD groups.

**Table 3:**
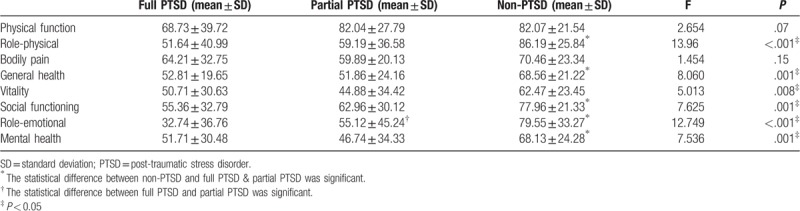
Analysis of health-related quality of life in transplant donors.

## 4 Discussion

Post-traumatic stress disorder is prevalent in pediatric transplant recipients.^[11]^ Previous literature has found the occurrence of PTSD to be associated with poor transplant outcomes, primary disease, educational status, and so on.^[11–14]^ However, evidence of PTSD in transplant donors has been limited. Therefore, this study conducted a detailed survey, involving the risk factors for PTSD and quality of life, in living donors for pediatric liver transplants, which may contribute to the prevention of PTSD.

In our survey, the incidences of full and partial PTSD in living donors were 12.1% and 31.1%, respectively, indicating that the development of postoperative full or partial PTSD is common in living donors for pediatric liver transplantation. Currently, most donors are patients or immediate relatives, and the transplantation may be high-risk and unpredictable. Therefore, based on this situation, transplant donors, especially parents, may experience great pressure, both physically and mentally. Young et al^[11]^ also reported that PTSD seemed to be most common in parents of recipients. Additionally, pediatric recipients are at an important stage of physical and mental development.^[13]^ Some complications may occur after liver transplant. Long-term use of immunosuppressants may also cause side effects to the body. Apart from this, donors are always anxious about recipients’ long-term survival rates and the psychological or social problems that may arise during their development. We also found that the postoperative health status of recipients was closely associated with the occurrence of PTSD in living donors. Thus, it can be seen that because of the particularity of the relationship between donors and recipients in pediatric liver transplantation, severe postoperative stress response or even PTSD are likely in donors.

This study found that higher educational status in donors was a protective factor for the occurrence of PTSD. In addition, the occurrence of PTSD was not only related to pathogenic factors or stress events^[15]^ but also to emotions, thoughts, behaviors, and physiological reactions due to individual differences.^[16]^ People with a higher educational status were generally more knowledgeable and found it easier to understand the process and cause of events. They could, therefore, adapt to sudden stress and manage psychological problems. Moreover, their interpersonal relationships enabled them to receive more support. Additionally, donors with a higher educational status were more likely to have a better job and receive higher compensation, which could have reduced their anxiety about economic burden and the stimulation of stress events.

Postsurgery stage was also associated with the development of PTSD. The severity of post-traumatic stress in donors decreased with time after surgery. Donors generally experienced the most stress and emotional instability in the initial months of the postoperative period. Annema et al^[17]^ found that in recipients, PTSD was most prevalent in the first year after liver transplantation, which was similar to our results on donors. Generally, stress in donors decreased with improved physical condition and disease resistance in recipients. With time, the occurrence of infection, acute rejection, and frequency of follow-up decreased, which may have contributed to the reduction of adverse stress and the alleviation of scene repetition. Therefore, this survey suggested the necessity of intervention measures to reduce stress stimulation and avoid the development of PTSD during the early post-transplant period.

Many studies have suggested that PTSD may lead to the reduction of quality of life in patients,^[18–24]^ and this is true in the case of organ transplantation as well. Young et al^[11]^ reported that PTSD seemed relatively common in parents of pediatric transplant recipients and their quality of life greatly reduced after pediatric bone marrow transplantation. We have previously reported in the case of adult liver transplantation, PTSD always leads to a poor quality of life for the recipients, especially from the mental perspective.^[6]^ However, this survey showed that donors also experience a lower quality of life, particularly in the domains of mental health, role-emotional, and social functioning. Therefore, PTSD can greatly lower the quality of life of donors. With the improvement of surgical techniques and immunological rejection management, number of cases of living organ transplantation will only increase, and rather rapidly. With the number of transplant donors also continually increasing, it is imperative that the questions of how to decrease stress and improve the quality of postoperative life be answered. Effective interventions must be administered to prevent the development of PTSD and improve HRQoL.

This study may contribute to the prevention of PTSD in pediatric living donors. First, to eliminate anxiety in donors, sufficient communication to enable them to fully understand the operation is essential, especially in donors who are parents of recipients and have lower educational status. Additionally, regular psychological counseling for donors is necessary, especially in the first 6 months after transplant. Different schemes can be decided upon based on donors’ educational backgrounds. Moreover, in our study, it was found that donors were more likely to develop PTSD when the recipients suffered severe complications. To avoid this situation, the further improvement of perioperative management, which may reduce the possibilities of severe surgical complications, is required.

Our study also had several limitations. First, as it was a single-center study, the generalizability of our results may be limited. Additionally, the sample size was relatively small owing to the low number of pediatric liver transplantations compared with adult transplantation. Apart from this, as compared with longitudinal data, cross-sectional data provide insufficient information to understand the changes in the severity of post-traumatic stress symptoms over time. Therefore, more related studies are needed. Despite these limitations, however, this is the first study to discuss the risk factors for PTSD and quality of life in living donors for pediatric liver transplantation, which may contribute to positive interventions for PTSD.

## 5 Conclusions

The occurrence of PTSD was common in living donors after pediatric liver transplantation. Those with lower educational status, who were donors to their own children, who were in the first 6 months after transplant, or who were involved in transplants where the recipients suffered severe complications were most likely to experience PTSD. Severity of post-traumatic stress symptoms was significantly associated with a poorer quality of life after transplantation.

## Author contributions

**Conceptualization:** Shuguang Jin.

**Data curation:** Yimao Zhang, Junxiang Wang, Kewei Li.

**Formal analysis:** Shuguang Jin, Bo Xiang.

**Investigation:** Yimao Zhang, Shuguang Jin.

**Methodology:** Jiaying Yang, Bing Huang.

**Project administration:** Yimao Zhang, Junxiang Wang.

**Resources:** Jichun Zhao.

**Software:** Yimao Zhang, Junxiang Wang, Wei Lai, Lunan Yan.

**Supervision:** Shuguang Jin.

**Validation:** Junxiang Wang.

**Visualization:** Shuguang Jin.

**Writing – original draft:** Yimao Zhang.

**Writing – review & editing:** Shuguang Jin.
